# Endogenous Circadian Rhythms in Plant Bioelectric Signals: Cross-Station Replication and Visitor-Driven Suppression in a Public Exhibition

**DOI:** 10.3390/biomimetics11060405

**Published:** 2026-06-08

**Authors:** Peter A. Gloor

**Affiliations:** 1 MIT System Design Management, Massachusetts Institute of Technology, Cambridge, MA 02142, USA; pgloor@mit.edu; 2 Cologne Institute for Information Systems, University of Cologne, 50923 Cologne, Germany; 3 Galaxy Advisors AG, 5000 Aarau, Switzerland

**Keywords:** plant bioelectrics, circadian rhythms, cosinor analysis, spectral analysis, *Primula vulgaris*, population-scale recording, biosensing, biomimetic sensing, mechanosensory coupling, naturalistic recording

## Abstract

We report a cross-station replication of endogenous circadian rhythms in plant bioelectric voltage, recorded continuously for 42 days at three independent sensor stations within a public science exhibition (Phänomena, Dietikon, Switzerland; March–April 2026). Three primrose (*Primula vulgaris*) stations were equipped with custom Biolingo bioelectric sensors (ESP32 + AD8232) and recorded autonomously through approximately 21,000 visitor interactions. We extracted DC-invariant spectral features from 5–10 s voltage windows (n = 78,431 quality-filtered files) and fitted two-stage cosinor models with bootstrap 95% confidence intervals. All three stations show a robust 24 h rhythm in the 1–5 Hz band power (bp_1–5_), with peak-to-trough amplitudes between 0.35× and 1.19× of mesor (R^2^_med_ 0.72–0.87). Acrophase varies across stations from 05:00 to 11:00 local time. Critically, the rhythm survives an overnight-only restriction (18:00–09:00, no visitors) at all three stations, ruling out visitor presence as the rhythm driver. The most visitor-intensive station (faces of museum visitors triggering an emotion-recognition installation) additionally shows a sharp daytime amplitude collapse coincident with the exhibition opening at 09:00, during the hours of sustained visitor presence. This temporal coincidence is consistent with—though not by itself proof of—the cardiovascular-mechanosensory coupling characterized at single-subject resolution in a companion study. We argue that bp_1–5_—the spectral band most directly related to plant action-potential activity—carries an endogenous circadian signal in *Primula vulgaris* and that this station-level signal co-varies with sustained nearby human presence in a manner consistent with frequency-selective mechanosensory coupling, although the observational design cannot establish this mechanism. From a biomimetic perspective, this suggests that the plant’s evolved bioelectric sensing apparatus might be leveraged as a live ambient biosensor for nearby human activity, complementing the more common biomimetic approach of replicating plant sensing in synthetic devices.

## 1. Introduction

Plant electrophysiology is positioned at the intersection of biology and engineering. From a biomimetic-design perspective, plants have inspired a generation of bio-engineered sensing and actuation systems, from plant-root-inspired robotic soil-monitoring probes [1,2] to soft-bending actuators that mimic the differential growth of climbing tendrils. A complementary line of work, sometimes called “phytosensing”, inverts the usual biomimetic flow: rather than build synthetic devices that mimic plant sensing, it uses the plant’s own evolved sensory apparatus as the sensor, instrumenting living plants with bioelectric front-ends to read out the signals they already produce in response to environmental and biological stimuli [3,4,5,6]. Both approaches share the assumption that plants are competent biological sensors whose evolved transduction mechanisms outperform synthetic alternatives in cost, robustness, and integrative capacity.

The present study extends the phytosensing line into population-scale and chronobiological territory, asking what the baseline endogenous behavior of such a living sensor looks like outside laboratory conditions and how that baseline is modulated by the very biological signals (human presence) that biomimetic deployments would seek to detect.

Plant circadian rhythms are well established at the molecular and physiological level. The CCA1/LHY/TOC1 transcriptional feedback loop in *Arabidopsis thaliana* drives roughly 24 h oscillations in gene expression [7], stomatal aperture [8], and photosynthetic capacity [9]. Less attention has been paid to whether these rhythms manifest in long-timescale plant bioelectric activity at a population scale and under naturalistic, uncontrolled environmental conditions. The handful of bioelectric circadian studies in the existing literature [10] have been laboratory-based, single-plant or small-N, and have used controlled light cycles—conditions that maximize statistical power but limit ecological validity.

A complementary research program has measured plant bioelectric voltage as a sensor for nearby biological events, including human emotional states [5,6]. In our previous work, we have demonstrated that plant voltage carries information about the movement, presence, and emotional state of nearby humans [5,6], across multiple plant species and sensor configurations. Most recently, we have shown that human cardiovascular activity couples to plant bioelectric oscillations through a frequency-selective mechanosensory pathway with an integration timescale (~34 s) strikingly similar to that of Venus-flytrap prey detection [11]. The companion study established this coupling at single-subject high resolution over a 26 h recording, characterizing how the plant responds within seconds to nearby human cardiovascular activity. The present paper addresses a complementary question at the opposite end of the timescale spectrum: what is the endogenous 24 h baseline on which such second-scale coupling rides? Coupling effects, after all, ride on top of whatever endogenous activity the plant itself generates—and that endogenous baseline has not been characterized at the timescales and high-throughput sampling densities that public-exhibition deployments now make accessible.

The present study uses a 42-day deployment of three independent bioelectric sensor stations within a public exhibition to address two questions. First, do plant voltage signals exhibit a 24 h rhythm that replicates across independent stations and survives the removal of visitor-presence hours? Second, can we detect the modulation of that endogenous rhythm by sustained human presence at a sufficiently visitor-intensive station to test the mechanosensory coupling hypothesis at a population scale?

We address these questions with cosinor analysis [12] of DC-invariant spectral features—standard deviation, interquartile range, two band powers (0–1 Hz and 1–5 Hz), and zero-crossing rate—extracted from 5–10 s WAV recordings sampled at 100 Hz. We use two-stage cosinor fitting (per-hour median for amplitude and phase estimation and full-N F-test for statistical significance) with bootstrap 95% confidence intervals on the per-hour profile. Per-day z-normalization decouples the circadian component from slow electrode drift. The overnight-only restriction (18:00–09:00) provides a within-dataset replication test: a rhythm that survives the no-visitor window cannot be a visitor-driven artifact.

The dataset is unusually large for a plant chronobiology study by virtue of being collected as a by-product of a public art-and-science exhibition rather than a controlled laboratory experiment. This trades some of the precision of laboratory work—light, temperature and humidity were not actively controlled; plants were occasionally replaced when visitor handling damaged the leaves; and visitor density varied substantially across days—for population-scale statistical power and ecological validity. A signal that survives all of this noise is plausibly real.

## 2. Related Work

This study sits at the intersection of three mature literatures: plant chronobiology, plant electrophysiology, and machine-learning approaches to non-invasive plant monitoring. We briefly review each research stream, with emphasis on the gap that motivates this paper, then situate our work relative to recent population-scale chronobiology approaches in human laboratory medicine.

### 2.1. Plant Chronobiology

Plant circadian rhythms have been recognized since de Mairan’s 1729 leaf-movement experiments, but the molecular basis was elucidated only over the past three decades. McClung [13] reviews the contemporary picture: the plant circadian oscillator is a highly complex network of interlocked transcriptional feedback loops, in which morning-expressed Circadian Clock Associated 1 (CCA1) and Late Elongated Hypocotyl (LHY) repress evening-expressed genes, including Timing of CAB Expression 1 (TOC1), which in turn represses CCA1/LHY [7]. This core loop interacts with multiple peripheral loops to produce the robust ~24 h rhythms that synchronize the plant’s physiology with the day–night cycle.

Outputs of the plant clock include both metabolic processes (photosynthesis and carbon partitioning) and movement (leaf folding and stomatal opening). Hassidim et al. [8] showed that CCA1 directly regulates stomatal aperture in a circadian-gated manner, and Dodd et al. [9] demonstrated that plants whose internal clock period matches the external photoperiod outperform mismatched plants in photosynthesis, growth, survival, and competitive advantage. Greenham and McClung [14] surveyed the broader output landscape, including how clock outputs feed back into stress-response and developmental pathways. The takeaway is that circadian regulation in plants is not peripheral; it is woven into the metabolic and physiological substrate that gives rise to the bioelectric activity we measure here.

Direct links between the circadian clock and plant bioelectric activity have been less extensively studied. Volkov et al. [15] demonstrated using charge-stimulation methods that the bioelectric circuits of Clivia miniata, Aloe vera, and Mimosa pudica show 24 h rhythms in input resistance and membrane potential that persist under constant light conditions, indicating endogenous control. Their data are compelling but were collected under laboratory conditions on a small number of plants. Whether such bioelectric rhythms manifest at a population scale under naturalistic conditions has not, to our knowledge, been previously addressed.

### 2.2. Plant Electrophysiology

Plant electrophysiology has its modern roots in the 19th-century work of Burdon-Sanderson and Darwin on Dionaea muscipula, and a robust contemporary literature has grown around two principal classes of plant electrical signal: action potentials (APs) and variation potentials (VPs). Fromm and Lautner [16] provide a foundational review of these signals and their physiological significance. Volkov et al. [10] characterized AP propagation kinetics in the canonical Venus-flytrap mechanosensory response, and Sukhov et al. [17] summarized how stimulus-specific information is encoded in the parameters of APs and VPs under various abiotic stressors. Toyota et al. [18] demonstrated long-distance calcium-based wound signaling in Arabidopsis at second-scale propagation speeds, expanding the catalogue of recognized electrical signal types in plants. Gallé et al. [19] reviewed the broader landscape of how environmental stimuli (temperature, wounding, light, and drought) trigger and shape plant electrical signals and how those signals in turn modulate physiological processes, including photosynthesis, transpiration, and respiration.

Two methodological challenges have shaped this literature. First, the timescales of interest span six orders of magnitude—from sub-second action potentials to circadian-period drifts—and no single sensor configuration captures all of them well. Most laboratory work uses bandpass filtering tuned to either the fast (APs and VPs) or slow (membrane drift) end of the spectrum, and few studies report behavior across the full spectrum simultaneously. Second, the practical difficulty of running stable long-duration recordings outside a Faraday cage, a grounded conductive enclosure that shields the high-impedance measurement from external electromagnetic interference, such as mains hum and radio-frequency pickup, has restricted electrophysiological work mostly to controlled-condition laboratories. Tran et al. [20] showed in 2019 that supervised machine learning could distinguish stress and non-stress conditions in plant electrical signals even when recordings were made outside a Faraday cage, opening the door to deployment in real cultivation environments.

### 2.3. Machine-Learning Approaches

Several groups have applied machine-learning techniques to plant electrophysiological data. Najdenovska et al. [3] trained classifiers on multi-channel electrical recordings from commercial tomato crops and identified a generalized stress signature distinguishing drought, nutrient-deficiency, and pest-infestation conditions from the baseline. Reissig et al. [21] used machine-learning classification to track tomato ripening stages from electrophysiological recordings, and González i Juclà et al. [4] subsequently applied deep-learning techniques to recover nitrogen-deficit signatures with cross-validated accuracy comparable to feature-engineered approaches. Kozlova et al. [22] reviewed the rapidly expanding “electrome” literature and laid out a framework for systematic electrophysiological phenotyping of plants. Together, these papers establish that plant electrical signals carry sufficient information to be useful for non-invasive crop monitoring and that modern statistical-learning techniques can extract that information with deployable accuracy. None of these studies, however, have examined the circadian timescale: their feature windows are short (typically 15 s to 30 min), and their classification targets are binary or categorical states rather than a continuous rhythmic structure.

### 2.4. Population Chronobiology and Naturalistic Recording

The chronobiology of human laboratory data has recently begun to embrace what Marques-Garcia et al. [23] term “population chronobiology with real-world data”: the extraction of circadian patterns from large pools of opportunistically collected, non-equidistant samples rather than from prospectively designed time series. Their work shows that even point-like data clouds, when aggregated across hundreds of thousands of patients, reveal robust 24 h rhythms in clinical chemistry markers under cosinor analysis. Cornelissen [12] earlier laid the methodological groundwork by reformulating cosinor analysis as a regression problem applicable to non-equidistant data and equipped with confidence intervals from least-squares theory. Fossion et al. [24] complemented this by surveying the broader landscape of irregular-rhythm quantification methods (Fourier, wavelet, and singular spectrum analysis) and the conditions under which each is most informative. The present study applies this population-chronobiology philosophy to plant bioelectric recordings collected naturalistically as a by-product of a public exhibition, extending it to a domain in which it has not previously been deployed.

### 2.5. The Position of the Present Study

Our prior work has used plant bioelectric voltage as a sensor for the presence and movement of nearby humans [6] and for their emotional state [5]. A companion manuscript [11] establishes a frequency-selective mechanosensory coupling pathway between human cardiovascular activity and plant bioelectric oscillations at the single-subject high-resolution level. The present paper complements that work by addressing what plant voltage looks like absent (or in the presence of) such coupling: what is the endogenous rhythmic baseline on which exogenous coupling effects ride? We frame this as a chronobiology question and use cosinor analysis to characterize the rhythm at a population scale across three independent recording stations, with the overnight-only no-visitor restriction as a within-dataset control for visitor-driven artifacts.

## 3. Materials and Methods

### 3.1. Exhibition Setup

The recordings analyzed in this study were collected at the Phänomena 2026 exhibition (Dietikon, Switzerland), a six-month public science exhibition that received approximately 500 visitors per day. Within the larger exhibition, the “Stille Signale” (“Silent Signals”) installation comprised four interactive stations, each pairing a *Primula vulgaris* plant with a different sensory modality: tactile (button-press), acoustic (Tibetan-bowl strike), photic (lamp), and emotional (visitor face captured by webcam and classified via HSEmotion). Three of these stations—lamp, sound, and emotion—produced sufficient quality-filtered data for circadian analysis and form the basis of this study. The touch station produced data at very different sampling rates and is excluded.

An extension of (iii) deserves separate mention. If the daytime suppression at the emotion station (Section 5.3) is real, then the visible 05:00 acrophase at that station may be a “recovery bounce”: a rebound oscillation following the period of damped daytime activity, which completes its rise overnight and peaks shortly before exhibition reopening. Under this interpretation, the emotion station’s “true” endogenous acrophase—absent visitor-driven suppression—would be later than 05:00 and might align more closely with the lamp station’s 11:00 peak. The overnight-only fit at the emotion station does shift the acrophase slightly later (5.3 h vs. 4.6 h all-hours; Table 1), consistent with the rebound-and-decay pattern, but the shift is small relative to the between-station phase spread, and we do not over-interpret it.

The exhibition was housed in a temporary inflatable balloon hall with no direct windows; ambient illumination came from artificial overhead lighting (on continuously from 09:00 to 17:00 and partially overnight) plus diffuse light permeating the hull during daytime. The hall was minimally heated: ambient temperature dropped to approximately 1–2 °C overnight and rose to 5–7 °C during the warmest part of the day during the recording window (early-to-mid March). This thermal regime drove the choice of *Primula vulgaris*: this temperate, frost-tolerant spring perennial remains physiologically active at temperatures that would induce chilling injury or metabolic dormancy in tropical species such as *Kalanchoe* and *Tradescantia*, which we have used in prior studies under temperate-laboratory conditions [11]. Pre-flowered specimens were also available locally and inexpensively (CHF 2.50 each at a Migros supermarket), and their leaves were robust enough to tolerate incidental visitor handling. We do not claim that *Primula* is biologically optimal for circadian electrophysiology; rather, it offers physiological viability under the cold ambient conditions of this specific deployment. Plants were watered manually from above approximately every three days, with watering events deliberately occurring during exhibition opening hours to avoid confounding the overnight recording window.

**Figure 1:**
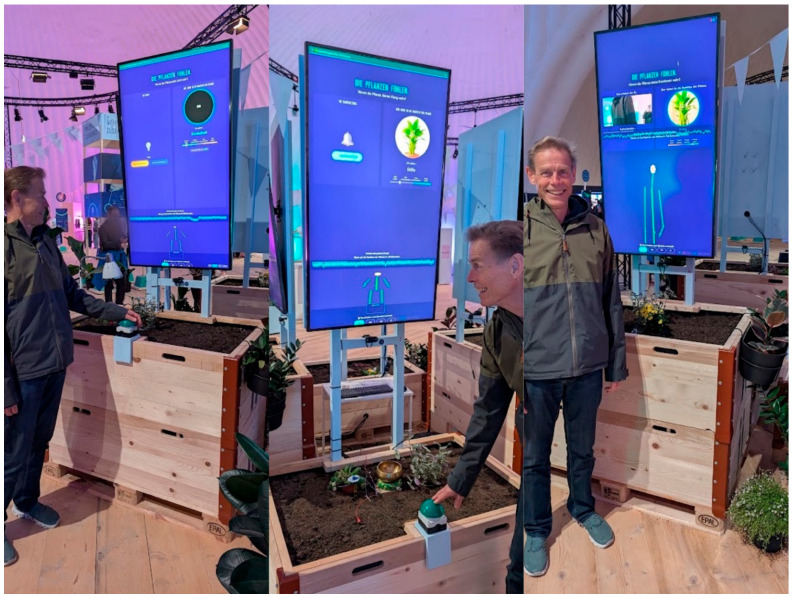
The three Phänomena exhibition stations analyzed in this study, photographed during operation. (**Left**): the lamp station (visitor pressing the green button illuminates the lamp). (**Middle)**: the sound station (Tibetan singing bowl visible at the foot of the screen; visitors trigger a bowl strike). (**Right**): the emotion station (visitor face captured by the screen-mounted webcam; HSEmotion classifies emotion in real time). Each station contains a planter box with one *Primula vulgaris* and an independent Biolingo bioelectric sensor unit; the gelled leaf electrode and the bare-metal soil electrode are not visible in the photographs. Photos: Phänomena exhibition, Dietikon, Switzerland, March 2026.

Each station (Figure 1) was equipped with a custom Biolingo sensor unit (galaxyadvisors AG, Aarau, Switzerland): an ESP32 microcontroller paired with an AD8232 single-lead bioelectric front-end, hand-soldered onto a custom PCB. The plant’s bioelectric voltage was measured between two contact points: one pre-gelled disposable Ag/AgCl disc electrode (the kind routinely used in clinical ECG) attached to a leaf, and a bare metal snap connector inserted directly into the moist pot soil, which served as the second lead via the soil’s intrinsic conductivity. The AD8232’s reference input (right-leg-drive) was connected to a third disc electrode left floating in the air; pilot work confirmed that the leaf-soil signal is preserved with the reference disconnected. Voltage was sampled at 100 Hz, transmitted over Wi-Fi via WebSocket to a Mac Mini M4 server, and saved as 5 to 10 s mono PCM WAV files (int16, 100 Hz nominal). Each WAV file was tagged with a timestamp, a station identifier, and—for the lamp, sound, and emotion stations—a label corresponding to the station’s stimulus state at the time of recording (lamp_on/off, strike/nostrike, or emotion_positive/negative). Over the 42-day recording window (3 March–20 April 2026), the three stations together produced 104,224 recordings.

Plants were replaced when leaf turgor visibly degraded (typically every 7–10 days). The unit of replication in this study is therefore the *station*—identical hardware in a fixed physical location—rather than the individual plant. Because each station is thus a continuous recording of one fixed sensor location through a succession of similar specimens rather than an uninterrupted recording of a single organism, the rhythm we characterize is best understood as a property of the station; the day-to-day stability across replacement events and the cross-station replication are what justify treating it as stable rather than an artefact of any single plant. Each station saw 2–4 different *Primula vulgaris* specimens during the 42 days. Staff were trained to detect torn or muddy electrodes and to reposition or replace plants as needed; nonetheless, some signal contamination is inevitable in a public-exhibition setting and is addressed in Section 3.3.

### 3.2. Feature Extraction

Each WAV file was treated as a single observation. We computed the following five features per file:

- std—sample standard deviation of the int16 voltage samples;
- iqr—interquartile range (75th–25th percentile);
- bp_0_1—power in the 0–1 Hz band, computed via Welch’s method (Hann window, 256-sample segment, and 50% overlap);
- bp_1_5—power in the 1–5 Hz band (same Welch parameters);
- zcr—zero-crossing rate of the DC-removed signal.

All features are DC-invariant by construction. This is essential because the AD8232 mid-rail bias drifts slowly over hours, and any feature sensitive to absolute voltage would conflate biological rhythm with electrode drift. We deliberately did not use Hjorth complexity, spectral entropy, or higher-order moments, which prior pilot work showed to be either unstable or dominated by 50 Hz mains contamination.

The 1–5 Hz band (bp_1–5_) is of particular interest. Plant action potentials in *Primula* and similar species span roughly 0.5–10 Hz [16]; the 1–5 Hz sub-band captures the spectral peak of action-potential activity while excluding both low-frequency drift (bp_0–1_) and high-frequency mains-related noise. The choice of this sub-band is further justified on three grounds. First, action and variation potentials in Primula and comparable species carry their dominant energy within roughly 0.5–10 Hz, so the 1–5 Hz window brackets the peak of biologically generated activity. Second, the adjacent 0–1 Hz region is dominated by slow electrode and membrane drift rather than active signaling, while the region above 5 Hz is increasingly intruded upon by mains harmonics and amplifier noise. Third, of the five DC-invariant features examined, the 1–5 Hz band power carried empirically the strongest and most reproducible 24 h rhythm, and the anti-phase zero-crossing-rate rhythm reported in Section 4.4 provides an independent, frequency-agnostic corroboration of the same diurnal structure.

### 3.3. Quality Filtering

Three filtering passes were applied to remove obviously corrupted recordings:

(i) WAVs with an int16 standard deviation below 50 (electrode disconnected) or with more than 95% of samples at the int16 rails (±32,767, saturated amplifier) were excluded. This removed approximately 25% of files and corresponds to events where visitors physically detached an electrode from the leaf.

(ii) WAVs with an std below 200 or with more than 50% of samples within 5% of either rail were flagged as ‘suspect’ and excluded from the primary analysis. Sensitivity analyses including these files did not materially change the cosinor estimates.

(iii) Visitors occasionally tore an electrode loose; staff sometimes reattached the electrode without cleaning the gel of accumulated soil. The signature of a muddy reattachment is a plausible-looking signal with reduced biological correlation. We do not have a per-file detector for this failure mode and rely on the cross-station replication argument as defense: contamination affecting a single station would not produce coordinated 24 h rhythms across three independent stations.

After filtering, 78,431 files entered the primary analysis (lamp: 26,584; sound: 14,386; emotion: 37,461). Within the overnight-only subset (hours 18:00–09:00), 42,683 files remained (lamp: 13,124; sound: 4183; emotion: 25,376).

### 3.4. Cosinor Analysis

We fitted a single-component cosinor model *y*(*t*) *= M* + *A* · *cos*(2*π*(*t* − *φ*)/*T*) + *ε* with T = 24 h to each (station and feature) combination. To produce stable amplitude (A) and acrophase (φ) estimates that are robust to heteroscedastic per-file noise, we used a two-stage procedure: (i) compute the median of the feature within each hour-of-day bin (24 medians per station-feature); (ii) least-squares-fit the cosinor parameters to those 24 medians, reporting amplitude in raw feature units and the relative amplitude rel_amp = 2A/|M| as a unit-free comparison metric. The R^2^ of this fit (denoted R^2^_med_) is reported as a goodness-of-fit measure for the rhythm shape.

Statistical significance was assessed separately by an F-test on the full per-file data: the model y = M + α·cos(2πt/24) + β·sin(2πt/24) was compared to the null y = M, and the F-statistic on the (α, β) pair was used to compute a *p*-value. This test is sensitive to N (and hence very small at our sample sizes), so we deliberately interpret that *p* in conjunction with rel_amp: rel_amp < 0.05× with *p* < 10^−100^ indicates ‘statistically significant but physiologically trivial’ and is not reported as evidence of a rhythm.

To decouple the circadian component from inter-day variability (electrode drift, plant replacement, and ambient temperature changes), we additionally fit cosinor on per-day z-scored features: for each day (*d*) and feature (*f*), z_d,t,f_ = (y_d,t,f_ − μ_d,f_)/σ_d,f_. This removes any day-level mean or scale variation and forces all variation to be within-day. A rhythm that appears in both raw and z-normalized analyses with consistent acrophase is strong evidence for a true circadian signal rather than an artifact of inter-day drift.

For each per-hour median in the cosinor input, we computed a 95% bootstrap confidence interval by resampling files within that hour 1000 times. The shaded bands in Figures 3–5 denote these intervals.

The analysis was repeated using only files recorded between 18:00 and 09:00 local time—a 15 h window during which the exhibition was closed and no visitors were present. This excludes the 09:00–18:00 h window when visitor presence could potentially drive the signal directly. A circadian rhythm that survives this restriction cannot be visitor-driven.

### 3.5. Software and Reproducibility

All analyses were implemented in Python 3.11 using NumPy 1.26, SciPy 1.11, pandas 2.1, and matplotlib 3.8. Welch periodogram computation used scipy.signal.welch with default Hann windowing. Cosinor fitting used numpy.linalg.lstsq on the cosine/sine basis. Bootstrap confidence intervals used numpy.random.default_rng with seed 42 for reproducibility. The complete analysis pipeline is available at https://github.com/pgloor/biolingo-plant-circadian (accessed 7 June 2026).

## 4. Results

### 4.1. Dataset Characteristics

**Figure 2:**
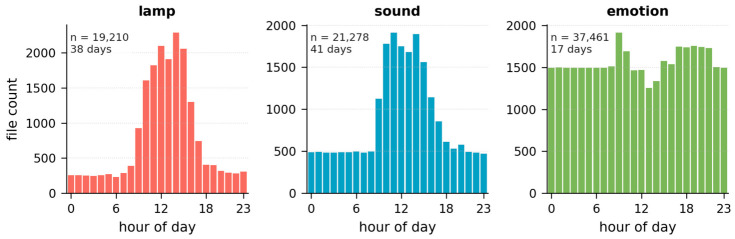
File-count distribution by hour of day across the three recording stations. Lamp and sound stations recorded continuously through the 42-day window; the emotion station recorded only during initial training phases of each application restart, producing fewer total recording hours but higher per-day density.

The 42-day recording window (3 March–20 April 2026) yielded 104,224 raw recordings across the three stations. After quality filtering (Section 3.3), 78,431 files (75.3%) entered the analysis. The distribution of recordings across the 24 h of the day was approximately uniform at the lamp and sound stations (which recorded continuously) and concentrated in 17 distinct active days at the emotion station, which only recorded during initial training phases of each application restart (a quirk of the emotion-station application architecture; see Section 5.4) (Figure 2). The recording-density imbalance is reflected in the per-station *n* in Table 1.

### 4.2. Circadian Rhythm in bp_1_5: Cross-Station Replication

Table 1 summarizes the cosinor fits for the 1–5 Hz band power across all three stations, for both the all-hours and overnight-only windows. The headline finding is the consistency of the bp_1–5_ rhythm across all three stations: rel_amp ranges from 0.35× (sound) to 1.19× (emotion) and R^2^_med_ from 0.72 to 0.87, all *p* < 10^−100^. The rhythm is visible in raw bp_1–5_ power, in z-normalized bp_1–5_, and—most critically—in the overnight-only subset for all three stations.

**Figure 3:**
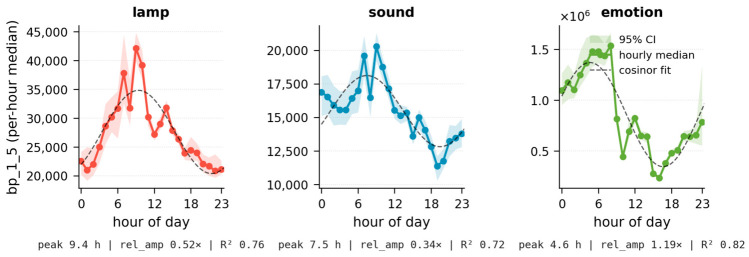
Per-hour median bp_1_5 profiles for the three recording stations across all 24 h of the day. Shaded bands: bootstrap 95% confidence intervals on the per-hour median (1000 resamples). Dashed line: cosinor fit. All three stations show clear periodicity well exceeding the confidence-interval width.

Figure 3 shows the per-hour median bp_1–5_ profile for all three stations over all hours of the day, with bootstrap 95% confidence intervals and the cosinor fit overlaid. Two observations are immediate. First, all three profiles are clearly periodic with peak-to-trough amplitudes well exceeding the 95% confidence intervals around the per-hour medians—this is not an artifact of N-driven significance. Second, the acrophases differ by approximately 6 h across the three stations: lamp peaks at 09:00–11:00, sound at ~07:00, and emotion at ~05:00. The phase variation is discussed in Section 5.2.

**Figure 4:**
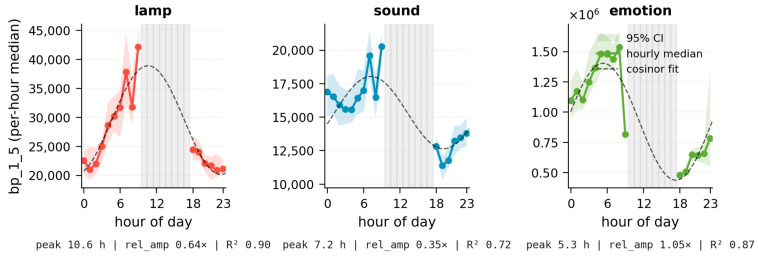
Overnight-only bp_1_5 circadian profiles, restricted to hours 18:00–09:00 when the exhibition was closed and no visitors were present. The grey shaded block marks the daytime hours excluded by this restriction. All three rhythms persist with amplitudes comparable to the all-hours analysis (Figure 3), confirming that the rhythm is not driven by visitor presence.

Figure 4 repeats this analysis with the overnight-only restriction (hours 18:00–09:00). All three rhythms persist with comparable amplitudes; the emotion station’s amplitude is slightly reduced (rel_amp 1.05× overnight vs. 1.19× all-hours) but still substantial. Acrophases shift by less than one hour at all three stations. This rules out visitor presence as the rhythm driver.

### 4.3. Daytime Suppression at the Emotion Station

**Figure 5:**
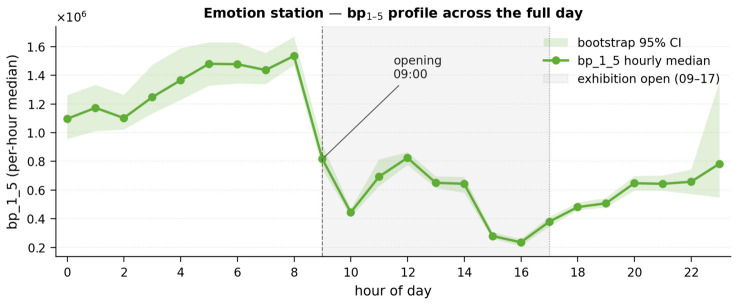
Detail of the bp_1_5 circadian profile at the emotion station, all hours. The green line is the per-hour median bp15 power and the green shaded band its bootstrap 95% confidence interval; the grey shaded block marks exhibition opening hours (09:00–17:00, when visitors were present), and the vertical dashed line at 09:00 marks opening time. Median power drops by ~50% in the hour following opening and remains suppressed through closing time at 17:00, recovering into the evening.

The emotion station shows a feature absent from the lamp and sound stations: a sharp daytime collapse of bp_1–5_ power coincident with exhibition opening at 09:00. Figure 5 shows the all-hours profile at the emotion station alone, with the 09:00 cliff edge highlighted. Median bp_1–5_ power drops from approximately 1.5 × 10^6^ at 08:00 to approximately 8 × 10^5^ at 10:00—a 50% reduction in one hour—and remains suppressed through 17:00 (closing time) before recovering through the evening to the overnight peak around 05:00.

**Figure 6:**
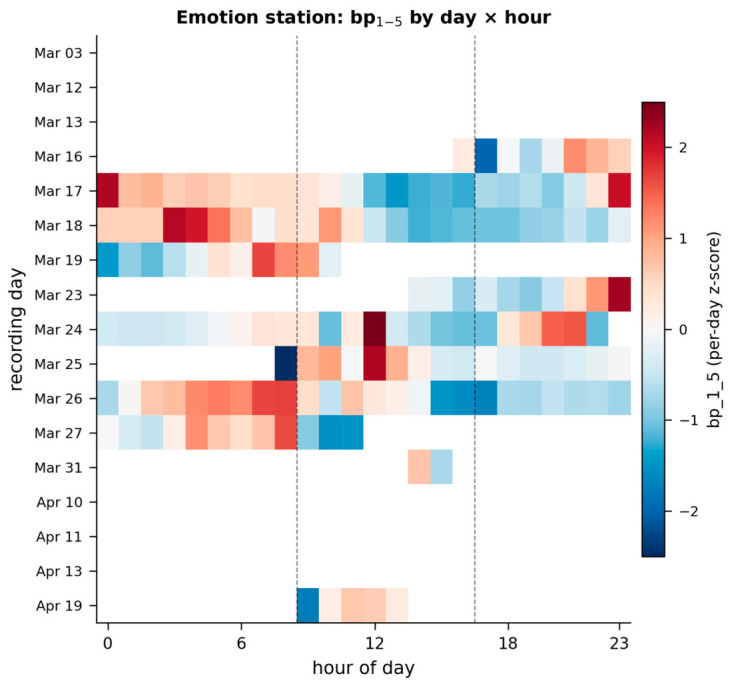
Day-by-day per-hour heatmap of bp_1_5 at the emotion station (per-day z-normalized). Each row is one recording day; each column is one hour of the day. Yellow = high relative power; dark blue = low. The vertical bands of low power between 09:00 and 17:00 are consistent across days, demonstrating the stability of the visitor-coincident suppression.

This pattern is not present at the lamp or sound stations. Figure 6 shows the day-by-day stability of the bp_1–5_ suppression: across the days the emotion station was active, the suppression onset is consistently between 09:00 and 10:00, with magnitude approximately invariant across days regardless of weekday or weather. The recovery curve through the evening and into the night is also consistent across days. The 09:00 onset is the time the exhibition opens to the public; the 17:00 trough corresponds to the end of opening hours.

### 4.4. Other Spectral Features

The std and iqr features—which integrate power across all frequencies, including the 1–5 Hz band where bp_1–5_ lives—show analogous but weaker rhythms. Their acrophases align with bp_1–5_ within 1–2 h, consistent with bp_1–5_ being the dominant contributor.

The bp_0_1 feature shows weaker rhythms with a smaller rel_amp (~0.3–0.6×) and lower R^2^_med_ (~0.65–0.80) than bp_1–5_, though the same acrophase. This is consistent with the 0–1 Hz band capturing slow electrode drift in addition to genuinely slow plant activity—introducing day-to-day baseline noise that washes out some of the circadian signal in the raw data and is partially recovered by per-day z-normalization.

The zero-crossing rate (zcr) shows a rhythm of opposite phase to the band powers, with acrophase at 16:00–18:00. This is consistent with zcr being inversely related to the dominant signal frequency: when the plant is in its high-power state (overnight), low-frequency content dominates, and the zero-crossing rate is low; when the signal is suppressed during visitor hours, relative noise increases, and the zero-crossing rate rises. zcr therefore offers an independent confirmation of the rhythm with the expected anti-phase relationship.

## 5. Discussion

### 5.1. Cross-Station Replication Establishes the Rhythm

The principal finding is the replication of a 24 h rhythm in the 1–5 Hz band power of plant bioelectric voltage across three independent recording stations, surviving an overnight-only window in which no visitors were present. The cross-station replication is the strongest single piece of evidence for the rhythm being a property of the recording stations rather than an artifact of any individual sensor, electrode, plant, or transient microenvironment. We are careful to distinguish station-level rhythmicity—a stable 24 h structure at a fixed instrumented location across a succession of plants—from individual-plant endogenous rhythmicity, which the present design cannot establish: because each station pooled 2–4 plants, part of the station-level rhythm may reflect time-of-day-structured environmental factors at that location rather than an intrinsic organismal clock. The hall temperature, for instance, swung from roughly 1–2 °C overnight to 5–7 °C during the day, and the overhead lighting followed a fixed daytime schedule; both co-vary with time of day, neither was logged continuously at the stations (Section 5.5), and either could contribute a 24 h-periodic component to the recordings. The overnight-persistence result argues against visitor presence as the rhythm driver, but it cannot by itself separate an intrinsic clock from other diurnally structured inputs; controlled single-plant recording with matched, logged conditions would be needed to establish organism-level endogeneity. Each station used independent hardware (separate ESP32 microcontroller, separate AD8232 amplifier, separate electrode set, and separate Wi-Fi link); they were located in physically separate parts of the exhibition with different lighting and visitor-flow patterns; and their plants were procured separately and replaced on independent schedules. A coordinated 24 h rhythm in bp_1–5_ across all three is therefore very unlikely to be a hardware artifact or single-plant idiosyncrasy.

The overnight-only test (Section 4.2, Figure 4) eliminates the most obvious confound: that visitor presence during the day directly drives the bp_1–5_ pattern. The rhythm is robust to this restriction at all three stations, with relative amplitudes in the overnight subset comparable to or larger than those in the all-hours data. The observation that R^2^_med_ actually improves at all stations under the overnight-only restriction—from 0.76 to 0.90 at lamp, 0.72 to 0.78 at sound, and 0.82 to 0.87 at emotion—reflects the elimination of the visitor-period non-sinusoidal cliff edge and confirms that the underlying biological rhythm is approximately sinusoidal.

### 5.2. Phase Variation Across Stations

The three stations show acrophases spanning approximately six hours: emotion peaks at 05:00, sound at 07:00, and lamp at 11:00. This is a substantial phase spread within a single species (*Primula vulgaris*) in a single building over a single season. Three classes of explanation should be considered.

(i) Differential entrainment from station-specific photic environments: The lamp station’s defining feature is a lamp that turns on and off in response to visitor button presses. The light is positioned for visual effect, not for photosynthesis, but is nonetheless visible to the plant during active hours. The sound and emotion stations have ambient and screen lighting respectively. Even modest inter-station differences in the photic regime could phase-shift entrainment, and a 6 h spread is plausible given that the plants were exposed to different light histories for 7–14 days each before measurement.

(ii) Plant-replacement schedules: Plants were replaced when their leaves became limp, on intervals of 7–10 days. A plant freshly arrived from the supplier’s greenhouse has a different recent light history (greenhouse photoperiod) than a plant that has been at the exhibition for two weeks. Different replacement timings could produce different ‘effective ages’ since last entrainment shock, and hence different acrophases.

(iii) Direct effects of the station’s characteristic stimulus on the plant’s clock: The emotion station, in particular, has the most sustained visitor proximity (visitors stand in front of the plant while their face is being analyzed by FER), the closest visitor-to-plant distance, and the most prolonged duration per visitor. If the modulation of plant electrophysiology by sustained nearby cardiovascular activity (companion paper [11]) extends to the entrainment of the plant’s clock—a possibility we cannot rule out—the emotion plant’s acrophase shift toward pre-dawn hours could be a long-term consequence of daytime suppression. The plant’s recovery oscillation may dominate during the unsuppressed overnight period, biasing the apparent acrophase earlier than it would be in the absence of visitor-driven damping.

We cannot decisively distinguish among these explanations with the present data. A controlled-condition replication—same species, identical photic regime across stations, and no visitor exposure—would be needed. The Phänomena dataset, with its uncontrolled inter-station differences, can only document the spread; it cannot localize its cause.

### 5.3. Daytime Suppression: Visitor-Coincident Modulation

The emotion station’s sharp 09:00 cliff edge is the secondary finding of this paper, and we interpret it cautiously as a population-scale pattern that is consistent with—but cannot, on its own, establish—the cardiovascular-mechanosensory coupling characterized at single-subject high resolution in our companion study [11]. Because the recording was made in an uncontrolled exhibition, the 09:00 effect coincides with several visitor-correlated covariates—screen and camera illumination, acoustic and structural vibration, electrical load, temperature and humidity, and systematic differences in visitors’ own state across stations—any of which could contribute to it; the observational design supports temporal coincidence rather than causal mechanistic attribution, and it is the companion single-subject study, not this dataset, that carries the mechanistic specificity. That study established, using lagged spectral mediation analysis on a single-subject 26 h dataset (n ≈ 93,000 1 Hz observations of co-located human and plant), that human cardiac activity (heart rate) fully mediates the valence→plant coupling specifically in the 44–75 s oscillation band of the plant’s STFT spectrum, with a 34 s integration delay reminiscent of Venus-flytrap mechanosensory integration.

The 1–5 Hz band power that we measure here in 5–10 s windows is the spectral slot occupied by plant action-potential activity, into which the 44–75 s oscillation modulation must propagate as an envelope effect: if HR-driven mechanosensory coupling damps slow oscillations in the 44–75 s band, the corresponding action-potential activity in the higher bp_1–5_ band will be modulated on a similar timescale. The 09:00 cliff edge is the population-scale signature of this damping: when the exhibition opens, ~50–100 visitors per hour begin standing in close (0.5–1 m) proximity to the plant for ~30–60 s each, providing approximately continuous ambient cardiac mechanical input to the plant. The plant’s slow oscillations are damped, the bp_1–5_ envelope drops, and the signal recovers only after closing time when the plant is left undisturbed.

The lamp and sound stations do not show this cliff edge, despite also being in the visitor flow. The difference is in the duration and proximity of visitor interaction. Lamp interactions are brief button presses—a visitor approaches, presses the button, watches the lamp turn on or off, and walks away—typically under 30 s and 1–2 m from the plant. Sound interactions are similar: trigger a bowl strike, listen for a few seconds, and move on. The emotion station, by contrast, requires a sustained interaction: visitors stand in front of the camera while the FER algorithm assesses their face, a process that takes 30–60 s and during which the visitor is typically 0.3–0.5 m from the plant. The emotion station produces approximately the right combination of sustained close-proximity cardiac exposure to drive mechanosensory damping; the lamp and sound stations do not.

This is consistent with—but does not by itself prove—the mechanosensory hypothesis. Alternative explanations include the emotion station’s visual environment differing from the others (the camera and screen produce different ambient illumination than a lamp or a Tibetan bowl) or the visitors’ subjective state differing systematically across stations (visitors who linger to be photographed are perhaps more emotionally engaged than those who briefly press a button). The single-subject mediation analysis in the companion paper provides the mechanistic specificity that this population-scale observation does not. Together, the two studies provide complementary evidence: the companion paper establishes the mechanism at high temporal resolution; this paper establishes that the macroscopic envelope of the same effect is observable at a population scale across many visitors.

A common concern with installations of this kind is that an apparent behavioral effect is actually a hardware artifact—the activation of an LCD screen, the powering up of a webcam, or a daytime increase in exhibition-floor electrical load. The exhibition design rules these out at all three stations. Each station was equipped with the same model of 55-inch LCD monitor and the same model of webcam, both running continuously throughout the 24 h cycle (the cameras additionally drove gesture-based language switching and so were active overnight as well as during exhibition hours). The monitors emit broadband visible light with a substantial blue-light component, and blue light is a known plant zeitgeber that could in principle entrain or modulate stomatal and photosynthetic gating. Because the monitors were on 24/7 and identical across stations, however, any monitor-driven photic effect should appear at all three stations equally and at all hours of the day equally. It does not. The cliff edge appears only at the emotion station and only during exhibition opening hours, which is the only place and time in which the visitor-interaction modality differs from the other stations. The hardware confound is therefore not merely “unlikely”; it is excluded by the experimental design.

What does differ across stations is the duration of visitor–plant proximity. The lamp station’s defining interaction is a button press that lights a lamp; visitors typically approach, press the button, observe, and walk away within 10 to 30 s. The sound station’s interaction is a Tibetan-bowl strike followed by a few seconds of listening; total visitor proximity is comparable, on the order of 5 to 30 s. The emotion station, by contrast, requires visitors to stand stationary in front of the camera while their facial expression is classified by the FER algorithm, a process that takes 30 to 60 s and during which the visitor is 0.3 to 0.5 m from the plant. This 30–60 s interaction window is the same timescale at which our companion paper [11] identified the plant’s frequency-selective mechanosensory integration: a 34 s STFT band carries the valence-mediated cardiac-coupling signal at single-subject high resolution. We interpret the population-scale cliff edge here as the macroscopic envelope of the same mechanosensory pathway: only the emotion station provides the sustained, close, and continuous proximity that allows for the integration window to fill, and only the emotion station shows the corresponding bp_1–5_ envelope drop. The lamp and sound stations’ briefer interactions fall below the integration threshold and produce no detectable damping.

A further temporal feature supports the visitor-interaction interpretation over a purely phototropic one: the recovery of bp_1–5_ power begins at approximately 17:00, the time the exhibition closes, rather than at a clock-driven solar-related time. A purely light-entrained circadian rhythm would be expected to follow either the building’s artificial-lighting schedule or (less likely, given the balloon-hall enclosure) a solar-related schedule; in either case the recovery should not coincide with a behavioral threshold, such as the departure of the last visitors. The fact that recovery tracks closing time tightens the link between the cliff edge and visitor presence.

### 5.4. Biomimetic Implications

The findings reported here extend a phytosensing approach to plant bioelectrics that is biomimetically distinct from the dominant plant-inspired-robotics line of research. The mainstream biomimetic literature on plant sensing has focused on replicating plant sensory mechanisms in synthetic devices: artificial roots that navigate soil chemistry [1,2], soft-bending actuators that mimic tendril circumnutation, and growth-driven robots that locomote by adding material at the tip in the manner of plant root meristems. The phytosensing approach, by contrast, takes the plant itself as the sensor and reads out its existing bioelectric signals via low-cost amplifier front-ends [5,6]. The two strategies are complementary: synthetic biomimetic sensors offer engineering tractability, customizability, and reproducibility; living phytosensors offer evolved sensitivity, distributed multimodal transduction, and a deployment cost dominated by the price of the plant rather than that of the transducer. Our 42-day deployment with −5 CHF specimens of *Primula vulgaris* demonstrates that the latter approach scales to public-environment deployment durations and visitor populations that would be prohibitively expensive with instrumented synthetic equivalents.

The mechanosensory damping observed at the emotion station, taken together with the 34 s integration window characterized in our companion paper [11], suggests a tentative design principle worth flagging for biomimetic engineers building proximity-sensing systems. The plant’s mechanosensory integration timescale of tens of seconds is in the same range as several other biological mechanosensory systems that have been studied in detail: the Venus flytrap’s 20–30 s action-potential summation [10], the mammalian baroreflex’s second-to-tens-of-seconds latency, and the slow-adapting cutaneous mechanoreceptors that distinguish sustained from transient touch. We do not claim a unifying principle across kingdoms; we observe only that biomimetic mechanosensors designed to discriminate sustained from transient stimuli might benefit from incorporating integration windows in this range and that engineers seeking to use plants as living presence sensors should expect a behavioral threshold around 10–30 s of sustained close proximity rather than instantaneous response.

The deployment described here also points toward a class of biomimetic applications in built environments. The exhibition setup is, in functional terms, a low-cost ambient biosensor array: three −60 CHF plants and approximately 300 CHF microcontroller and amplifier hardware per station, distributed across a public space and capable of detecting visitor presence and (per the companion study) cardiovascular activity at a population scale, without instrumenting individual visitors and without privacy-sensitive biometric capture. The 25–75% relative-amplitude rhythms we observe across stations set a clear threshold above which a deviation could be flagged as anomalous. Whether such systems deliver useful signal-to-noise ratios in real built environments remains to be tested, but the present work establishes the baseline measurement framework on which such biomimetic infrastructure could be built.

### 5.5. Limitations

This study has several important limitations:

- Single species: All three stations used *Primula vulgaris*. The rhythm we describe may be specific to this species or to its current physiological state in early spring. Replication in other species (Kalanchoe, lettuce, and basil—species we have used in prior studies) would strengthen the generality claim. Species-specificity also matters for the mechanosensory interpretation in Section 5.3: different species have different mechanosensory thresholds and integration timescales (the Venus-flytrap-like 34 s integration in our companion paper [11] was measured in Kalanchoe, not Primula), so the magnitude of the cliff edge here may not generalize directly to other species at other distances.
- Plant replacement: As noted, individual plants were swapped 2–4 times per station during the recording period, on schedules driven by leaf turgor rather than experimental design. Each station is therefore not strictly a longitudinal recording of a single plant but a longitudinal recording of a fixed sensor location with a sequence of similar plants. The cross-station replication argument and the within-station day-to-day stability (Figure 6) argue that this does not invalidate the rhythm finding.
- Electrode contamination: We have no per-file detector for muddy electrode reattachment, only for fully detached or saturated states. Sensitivity analyses (excluding the lowest-amplitude 25% of files) did not change the rhythm estimates substantially, but a contaminated subset that produces plausible-looking but biologically uncorrelated voltage cannot be definitively excluded. The replication across three independent stations is the main defense against this concern.
- Single season, single location: Recording spanned 3 March–20 April 2026 in a single building in Dietikon, Switzerland (47° N). Different photoperiods (later spring and summer) and different latitudes might produce different acrophases or different relative amplitudes.
- Recording-density imbalance: The emotion station produced 37,461 quality-filtered files concentrated in 17 distinct recording days, while the lamp station produced 26,584 files spread across 38 days. This is an artifact of the emotion application’s architecture (recording only during initial training phases) and means that the emotion station’s rhythm estimate is based on richer per-day sampling but fewer days. The day-by-day heatmap (Figure 6) shows that the rhythm is stable across the days that are sampled, but a longer continuous recording at the emotion station would be needed to detect any slow drift.
- Visitor density was not directly measured: The total daily visitor count is approximately 500 (exhibition-wide average), but hour-by-hour density at each individual station is not available. We use exhibition opening hours (09:00–17:00) as a binary proxy for visitor presence. Since the bp_1_5 cliff edge at 09:00 is itself sharp and consistent across days, this binary proxy is sufficient for our claims.
- Environmental covariates were not directly logged: Although the qualitative characteristics of the balloon-hall environment are known (no direct daylight, artificial overhead lighting on a fixed schedule, and an ambient temperature of 1–2 °C overnight rising to 5–7 °C during daytime), we did not log temperature, humidity, or irradiance directly at the recording stations. Small day-to-day variations in any of these could contribute to the inter-day variability visible in Figure 6. A future deployment would log these covariates alongside the bioelectric signal.

## 6. Conclusions

We have demonstrated that the 1–5 Hz band power of plant bioelectric voltage exhibits a robust 24 h rhythm in *Primula vulgaris*, replicated across three independent sensor stations in a public exhibition, surviving an overnight-only restriction that excludes visitor presence. The rhythm has peak-to-trough amplitudes between 0.35× and 1.19× of mesor across stations, with R^2^_med_ between 0.72 and 0.87. Acrophases vary across stations from 05:00 to 11:00, suggesting station-specific entrainment effects that warrant controlled-condition follow-up. The most visitor-intensive station additionally shows a sharp daytime suppression of the rhythm, with onset at 09:00 (exhibition opening) and recovery after 17:00 (exhibition closing), consistent with the cardiovascular-mechanosensory coupling reported in our companion study at single-subject high resolution [11].

Three methodological contributions emerge. First, the use of DC-invariant features (std, iqr, bp_0–1_, bp_1–5_, and zcr) avoids the slow-drift confound that plagues raw-voltage circadian analyses. Second, the two-stage cosinor procedure (per-hour median for amplitude/phase and full-N F-test for significance) decouples the ‘shape’ question (is the per-hour profile sinusoidal?) from the ‘detectability’ question (is the amplitude statistically resolvable above per-file noise?), which are too often conflated in the chronobiology literature. Third, the within-dataset overnight-only restriction provides a powerful sanity check that any externally induced rhythm mimic must fail.

Future work should: (i) replicate the rhythm in additional plant species under controlled-condition laboratory settings, ideally with simultaneous direct measurement of stomatal conductance to anchor the bioelectric rhythm to a known physiological correlate; (ii) test the mechanosensory hypothesis at a population scale through a controlled experiment in which a subset of stations is shielded from visitor cardiac activity (Faraday cage or vibration isolation); and (iii) investigate whether the cross-station phase variation reflects entrainment differences or station-specific stimulus effects through a controlled multi-station deployment with matched photic regimes.

Plants have circadian rhythms; this is not news. That those rhythms are detectable at a population scale through opportunistic recording in a public exhibition, that they co-vary with the presence and cardiovascular activity of nearby humans in a manner consistent with mechanosensory coupling, and that all of this is observable using inexpensive open-source bioelectric sensors—this we offer as the contribution of the present study.

Beyond plant chronobiology proper, these findings position the live plant as a promising candidate biomimetic sensor for human-presence and human-cardiovascular signals in naturalistic settings. The biomimetic vision the work points toward is a built environment in which existing ornamental plants serve as a distributed ambient biosensing layer for human activity, complementing rather than replacing synthetic sensor infrastructure. The deployment economics of phytosensing—supermarket-priced plants and microcontrollers under 50 CHF—make multi-month, multi-station, and public-environment deployments tractable today; what the present work adds is a characterized endogenous baseline against which human-driven deviations from that baseline can be detected and quantified.

## Figures and Tables

**Table 1:** Cosinor fits to the bp_1_5 band across three stations: raw all-hours data and the overnight-only (18:00–09:00) replication subset.

Station	Window	n	rel_amp	peak_h	R^2^_med	*p*-Value
lamp	all hours	26,584	0.52×	9.4	0.76	<10^−300^
lamp	overnight only	13,124	0.64×	10.7	0.90	<10^−300^
sound	all hours	14,386	0.35×	7.2	0.72	<10^−300^
sound	overnight only	4183	0.42×	7.5	0.78	<10^−150^
emotion	all hours	37,461	1.19×	4.6	0.82	<10^−300^
emotion	overnight only	25,376	1.05×	5.3	0.87	2 × 10^−217^

rel_amp is the peak-to-trough amplitude of the cosinor fit divided by |mesor|; values of 1.0× mean the peak is twice the trough. peak_h is the acrophase in local (Central European) time. R^2^_med is the coefficient of determination of the cosinor fit to the 24 per-hour medians. *p*-values are F-test on the full per-file data and are sensitive to N.

## Data Availability

The datasets generated and analyzed during the current study are available from figshare at https://doi.org/10.6084/m9.figshare.32180721 (accessed on 5 May 2026).
